# Modifiability of residual force depression in single muscle fibers following uphill and downhill training in rats

**DOI:** 10.14814/phy2.14725

**Published:** 2021-01-27

**Authors:** Parastoo Mashouri, Jackey Chen, Alex M. Noonan, Stephen H. M. Brown, Geoffrey A. Power

**Affiliations:** ^1^ Department of Human Health and Nutritional Sciences College of Biological Sciences University of Guelph Guelph Ontario Canada

**Keywords:** actin, concentric, muscle, muscle fiber, residual force depression, rodent, speed, work of shortening

## Abstract

Following active muscle shortening, steady‐state isometric force is less than a purely isometric contraction at the same muscle length and level of activation; this is known as residual force depression (rFD). It is unknown whether rFD at the single muscle fiber level can be modified via training. Here we investigated whether rFD in single muscle fibers is modifiable through downhill and uphill running in the extensor digitorum longus (EDL) and soleus (SOL) muscles in rats. Rats were run uphill or downhill 5 days/week for 4 weeks. After muscles were dissected and chemically permeabilized, single fibers were tied between a length controller and force transducer, transferred to an activating solution, with ATP and pCa of 4.2 for mechanical testing. rFD was quantified after active fiber shortening from an average sarcomere length (SL) of 3.1–2.5 µm at a relative speed of 0.15 fiber lengths/s (slow) and 0.6 fiber lengths/s (fast). rFD was calculated as the difference in force (normalized to cross‐sectional area) during the isometric steady‐state phase following active shortening and the purely isometric contraction. In addition to rFD, mechanical work of shortening and stiffness depression were also calculated. rFD was present for both the EDL (6–15%) and SOL (1–2%) muscles, with no effect of training. rFD was greater for the EDL than SOL which closely corresponded to the greater stiffness depression in the EDL, indicating a greater inhibition of cross‐bridge attachments. These results indicate that while rFD was observed, training did not appear to alter this intrinsic history‐dependent property of single muscle fibers.

## Graphical abstract

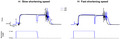

## 1 INTRODUCTION

Residual force depression (rFD) is the decrease in isometric steady‐state force following active shortening in comparison to a reference isometric contraction (ISO) performed at the same muscle length and level of activation (Abbott & Aubert, 1952; Chen et al., 2019; Chen & Power, 2019; Herzog, 2004; Rassier & Herzog, 2004). Residual force depression is an intrinsic property of muscle and has been investigated for decades; however the exact mechanisms are still under debate. To gain additional insights about rFD and its physiological applications, investigating the modifiability and trainability of rFD at the single muscle fiber level is necessary.

A commonly proposed mechanism for rFD is an actin angular deformation that occurs during active muscle shortening, which inhibits the binding of the myosin heads to actin (Joumaa et al., 2018; Maréchal & Plaghki, 1979), leading to fewer attached cross‐bridges and reduced force output (Joumaa et al., 2018). The magnitude of rFD is dependent on the amount of work performed during the active shortening phase (Corr & Herzog, 2005; Dargeviciute et al., 2013; Herzog & Leonard, 1997; Herzog et al., 2000; Lee et al., 2000; Leonard & Herzog, 2005; Minozzo & Rassier, 2013). Since work is the product of force and displacement, rFD could be modified by any adaptation at the single muscle fiber level that changes the amount of force being produced or the length a fiber is shortened.

Although rFD has been studied extensively over the last six decades, little attention has been given to how fiber type influences rFD. As described by Bottinelli et al. (1991), fast (MHC IIa, IIb, IIx) and slow muscle fibers (MHC I) have distinct force‐velocity properties, which dictates the amount of force and work they can perform for a given shortening velocity (Bottinelli et al., 1991; Joumaa et al., 2015). Only two studies have investigated the effect of fiber type on rFD in single muscle fibers (Joumaa et al., 2015; Pinnell et al., 2019). Joumaa et al. (2015) showed greater rFD in type II (rabbit psoas) single fibers as compared with type I (rabbit soleus) when shortening at an absolute speed. As expected, when shortened at an absolute speed, fast type II fibers performed significantly more work, and hence greater rFD, as compared with slow type I fibers. However, when the shortening speed was normalized to maximal shortening velocity, rFD was independent of fiber type. Similarly, Pinnell et al. (2019) showed rFD was present, and not different across fiber types, in human single muscle fibers biopsied from the vastus lateralis.

Training could potentially modify rFD by increasing single muscle fiber force production capacity and thus work of shortening. The only studies that have investigated the chronic modifiability of rFD following training were two in‐vivo studies testing humans (Chen & Power, 2019; Hinks et al., 2020), and an in‐vitro study in rodents (Chen et al., 2020). rFD of the ankle dorsiflexors was not modifiable at the whole human level following 4 weeks of concentric and eccentric resistance training (Chen & Power, 2019), nor 8 weeks of isometric training biased to a long or short muscle tendon unit length (Hinks et al., 2020). Similarly, rFD does not seem to be modifiable for the SOL and EDL following uphill and downhill training in rats despite the marked difference between the uphill and downhill groups for serial sarcomere number, respectively (Chen et al., 2020). It was thought that by altering the number of sarcomeres in series, each sarcomere would be shortened more or less which would alter work of shortening, presumably leading to differential adaptations to rFD (Chen et al., 2020). It is critical to note, although these findings are important additions to the literature, the authors are unable to tease out whether their null findings are due to scaling issues from the cellular to the whole muscle level, or other muscular components that contribute to force production. While there does not appear to be an effect of training on rFD at the whole muscle level, investigating alterations to these mechanical properties at the cellular level allows us to understand whether this property is intrinsically modifiable or not, which is imperative to our understanding of rFD and its physiological application.

This study aims to investigate whether rFD is modifiable through uphill and downhill training at the single muscle fiber level in male Sprague‐Dawley rats. Based on the available literature at the whole muscle level it was hypothesized that (i) there will be no change in rFD following training at the single muscle fiber level. (ii) Fibers from the fast‐type EDL muscle will experience more rFD owing to their ability to produce more work during active shortening compared to fibers from the slow‐type SOL muscle. (iii) Active shortening performed at a slow speed will result in greater rFD compared to a fast speed due to more work being performed during a slow compared to a fast shortening contraction.

## 2 METHODS

### 2.1 Animals

Thirty‐one male CD^®^ Sprague‐Dawley IGS rats (sacrificial age: 18.8 ± 0.28 weeks, mass: 523.6 ± 11.0 g) were obtained (Charles River Laboratories) for study, with approval from the Animal Care Committee of the University of Guelph. Rats were housed in groups of three, with a maximum of two groups at any given time, and free fed a Teklad global 18% protein rodent diet (Envigo) and water. After a week of acclimation to the new housing conditions, each rat was familiarized with running and assigned to one of three experimental groups: uphill running, downhill running, and sedentary control (i.e., no running intervention). Following the 20 days of exercise, rats recovered for 72 hr before sacrifice via CO_2_ asphyxiation followed by cervical dislocation, for experimental testing.

### 2.2 Training protocol

One week prior to training, rats were familiarized with treadmill running (on a 0° grade). Rats in the exercise intervention groups (i.e., uphill/downhill running) were run 5 days/week (i.e., Mon‐Fri) on an EXER 3/6 animal treadmill (Columbus Instruments) set to a 15° incline or decline for 20 training days, over a 4‐week period. Training sessions lasted 15 min on the first day and the daily duration was increased by 5 min/day, up to the target 35 min (by the fifth training day) for the remainder of the training period. At the start of each training session, rats were first introduced to a walking speed of 10 m/min, which was gradually increased to the 16 m/min target running speed, at a rate of 1 m every min. Rats were provided with 2 min of rest after each 5 min bout.

### 2.3 Solutions

The dissecting solution was composed of the following (in mM): K‐proprionate (250), Imidazole (40), EGTA (10), MgCl_2_·6H_2_O (4), Na_2_H_2_ATP (2), H_2_O. The storage solution was composed of K‐proprionate (250), Imidazole (40), EGTA (10), MgCl_2_·6H_2_O (4), Na_2_H_2_ATP (2), glycerol (50% of total volume after transfer to 50:50 dissecting:glycerol solution), as well as leupeptin (Sigma) protease inhibitors. The skinning solution with Brij 58 was composed of K‐proprionate (250), Imidazole (40), EGTA (10), MgCl_2_·6H_2_O (4), 1 g of Brij 58 (0.5% w/v). The relaxing solution: Imidazole (59.4), K.MSA (86), Ca(MSA)2 (0.13), Mg(MSA)_2_ (10.8), K3EGTA (5.5), KH_2_PO_4_ (1), H_2_O, Leupeptin (0.05), Na_2_ATP (5.1), as well as leupeptin (Sigma) protease inhibitors. The pre‐activating solution: KPr (185), MOPS (20), Mg(CH_3_COOH)_2_ (2.5), ATP (2.5). Activating solution: Ca^2+^ (15.11), Mg (6.93), EGTA (15), MOPS (80), ATP (5), CP (15), K (43.27), Na (13.09), H_2_O. All solutions were adjusted to a pH of 7.0 with the appropriate acid (HCl) or base (Tris).

### 2.4 Tissue preparation

The soleus (SOL) and extensor digitorum longus (EDL) muscles were harvested from the rats following sacrifice and were placed in chilled dissecting solution. The tissues were then placed in a silicone elastomer‐plated petri dish with the same solution and several bundles of approximately 0.5–1 mm in width and 3 mm in length were dissected out and transferred to a 2.5 ml tube containing 2.5 ml of chilled skinning solution added for 30 min and maintained on ice. Gentle agitation was performed to ensure all bundles were permeabilized equally in the skinning solution, ensuring all bundles were submerged at all times. The bundles were then washed with fresh, chilled, dissecting solution and gently agitated to remove any remaining skinning solution and stored in a separate tube containing storage solution. The bundles were incubated for 24 hr at 4°C. 0.6 ml tubes were prepared with fresh storage solution and the bundles were placed separately in each tube and placed in −80°C, until mechanical testing began. Our method of tissue preparation was adapted from Roche et al., 2015.

### 2.5 Mechanical testing and force measurements

Single fibers were dissected from the bundles in relaxing solution then transferred into a temperature‐controlled chamber (16°C) filled with relaxing solution and tied with nylon suture knots between a force transducer (model 403A; Aurora Scientific) and a length controller (model 322C; Aurora Scientific). The average sarcomere length (SL) was measured using a high‐speed camera (Aurora Scientific). Before starting the testing protocol, a ‘fitness’ contraction was performed in pCa 4.2 to ensure the ties were not loose and the fiber was in good condition. After the fitness test, SL was re‐checked and, if necessary, re‐adjusted to 2.5 μm. To begin muscle contraction, the fibers were transferred to a pre‐activating solution with ATP and then transferred to an activating solution, with ATP and a pCa of 4.2. Fiber length (L_0_) was recorded, and fiber diameter was measured at three different points along the fiber using a reticule on the microscope in relaxing solution. Micro‐manipulators on the stage were used to move the fiber into place along the ruler, and the average of the fiber diameters was used to calculate cross‐sectional area (CSA) assuming circularity. Two separate sets of tests were run on each fiber and the order of contraction was always performed in the following sequence: rFD (slow), ISO, rFD (fast), ISO. This order biases our results to lower rFD values by accounting for any fatigue/damage that could accumulate over time. If rFD is present, we can be sure it is owing to the active shortening, not some extraneous factor.

**FIGURE 1:**
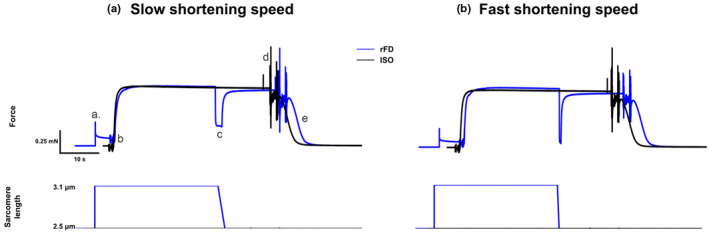
Representative force tracing for a (a) slow active shortening (0.15 Lo/s) and (b) fast active shortening (0.6 Lo/s) for the rFD (blue) and isometric reference (black) conditions. (a) passive stretch of the reference isometric condition from the 2.5 to 3.1 µm. (b) activation in both conditions. (c) active shortening from 3.1 to 2.5 µm in the force depression condition. (d) instantaneous stiffness test in both conditions. (e) Deactivation in both conditions. Noise represents changing of baths. The order of contraction was always performed in the following sequence: rFD (slow), ISO, rFD (fast), ISO

The fibers were set to a SL of 2.5 μm and were held in pre‐activating solution (reduced Ca^2+^ buffering capacity) for 30 s, where a passive stretch to 3.1 μm was performed. 5 s following the passive stretch, the fibers were activated (Ca^2+^ and high ATP) and were given 25 s to reach steady state, before being actively shortened to a SL of 2.5 μm at a speed of 0.15 L_0_/s (slow shortening contraction) or 0.6 L_0_/s (fast shortening contraction) (Figure 1a,b). 10 s following the active shortening, a stiffness measurement was taken by stretching by 0.3% of L_0_ at a speed of 3 L_0_/s, after which the fibers were deactivated by being moved to relaxing solution. For the ISO, the fibers were set to a SL of 2.5 μm and activated for the same duration as the rFD protocol and a stiffness measurement was taken at the same time following activation as the rFD protocol. All force measures were adjusted for resting passive tension by subtracting the average baseline value for the first 50 ms of the trial in the relaxing solution, thus for all contractions, active force is reported. All force values were normalized to CSA.

### 2.6 Fiber exclusion

Data points that were more than 2 *SD* away from the mean were excluded from analysis. *N* = 54 fibers from the EDL were tested and *N* = 55 fibers from the SOL. For analysis, *N* = 4 fibers and *N* = 5 fibers were excluded for the EDL and SOL, respectively. Each fiber underwent 2 rFD protocols and 2 ISO protocols: rFD (slow), ISO, rFD (fast), ISO. Thus, a maximum of *N* = 108 and *N* = 110 rFD/ISO tests were performed for EDL and SOL, respectively. Any fiber that slipped or ripped before the completion of its corresponding ISO test was removed from the testing apparatus.

### 2.7 Muscle type

The EDL muscle is 97% type II fibers and the SOL muscle is known to be >95% type I fibers in Sprague‐Dawley rats. Therefore, single muscle fibers were not fiber‐typed based on myosin heavy chain (MHC) content. (Ariano et al., 1973; Bloemberg & Quadrilatero, 2012; Collins et al., 2017; Eng et al., 2008; Ianuzzo et al., 1977; Nwoye et al., 1982; Ranatunga & Thomas, 1990; Rubinstein & Kelly, 1978; Wigston & English, 1992;). Given the fiber type distributions of these muscles, for our sample, this means that there was less than 2 out of the 54 EDL fibers tested being type I fibers and less than 3 out of the 55 SOL fibers tested being type II fibers. Based on these data, an assumption was made that fibers tested from the EDL muscle were fast type fibers, and fibers tested from the SOL muscle were slow type fibers. Two speeds (slow, 0.15 L_0_/s; fast, 0.6 L_0_/s) were chosen to embolden any discrepancies between the two muscle types, and all individual data points are presented in the graphs.

### 2.8 Analysis

All force values were normalized to CSA before calculations of the dependent measures were completed (i.e., absolute rFD, normalized isometric force, etc.). Transient aspects of force: Mechanical work of shortening in the rFD trial was calculated as the area under the force‐length curve during the shortening phase. Instantaneous stiffness was calculated as the difference between peak force during the stretch and the average force over the 500 ms prior to the stretch, divided by the change in fiber length induced by the stretch. %stiffness was calculated as the percent difference between the absolute stiffness values in the rFD condition compared to the ISO condition. For the rFD and the ISO values, force was reported as the 500 ms average prior to the instantaneous stiffness test at the same time following activation for all conditions. ISO and all rFD forces were compared at the same final muscle length and level of activation. rFD was calculated as the percent difference between the average force in the rFD condition compared to the ISO condition.

### 2.9 Statistical analysis

To confirm that rFD was indeed present for both muscles and all groups, two‐tailed paired t‐tests were performed. A 2‐way ANOVA was run to assess differences between ISO force production across muscles and groups, with ISO force (normalized to CSA) as the dependent variable. A 3‐way ANOVA was run to address whether %rFD, absolute rFD, %stiffness and specific work of shortening differed across groups, muscles and speeds. For both ANOVAs a Holm‐Sidak Post Hoc test was performed. Linear regressions were performed between the change in instantaneous stiffness and %rFD between the ISO and the rFD condition, and between specific work of shortening and absolute rFD.

## 3 RESULTS

### 3.1 Normalized isometric force and specific work of shortening

**FIGURE 2:**
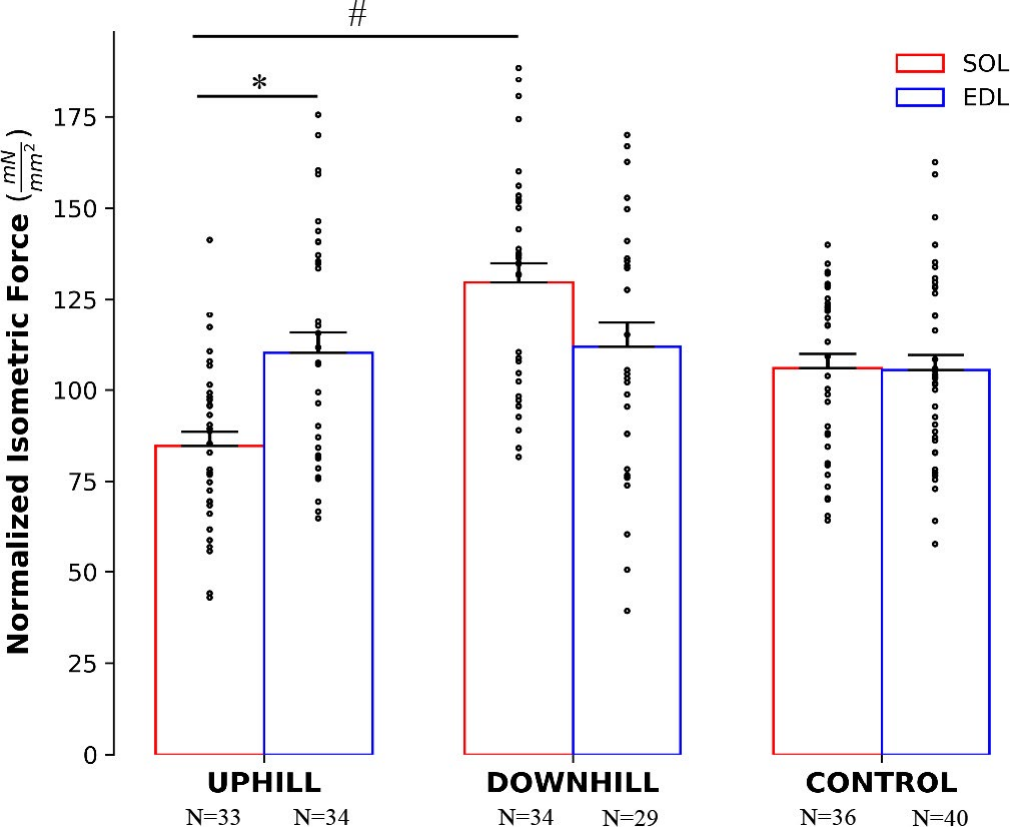
Normalized isometric force in the uphill, downhill, and control groups. The red bars represent the SOL muscle and the blue bars represent the EDL muscle. Within the uphill group, the EDL produced 18.9% more force than the SOL (*p* < .001). Within the SOL, the uphill group produced 37.4% less force as compared with the downhill group (*p* = .008). Mean ± SEM. ^*^Effect of muscle. ^#^Effect of group

**FIGURE 3:**
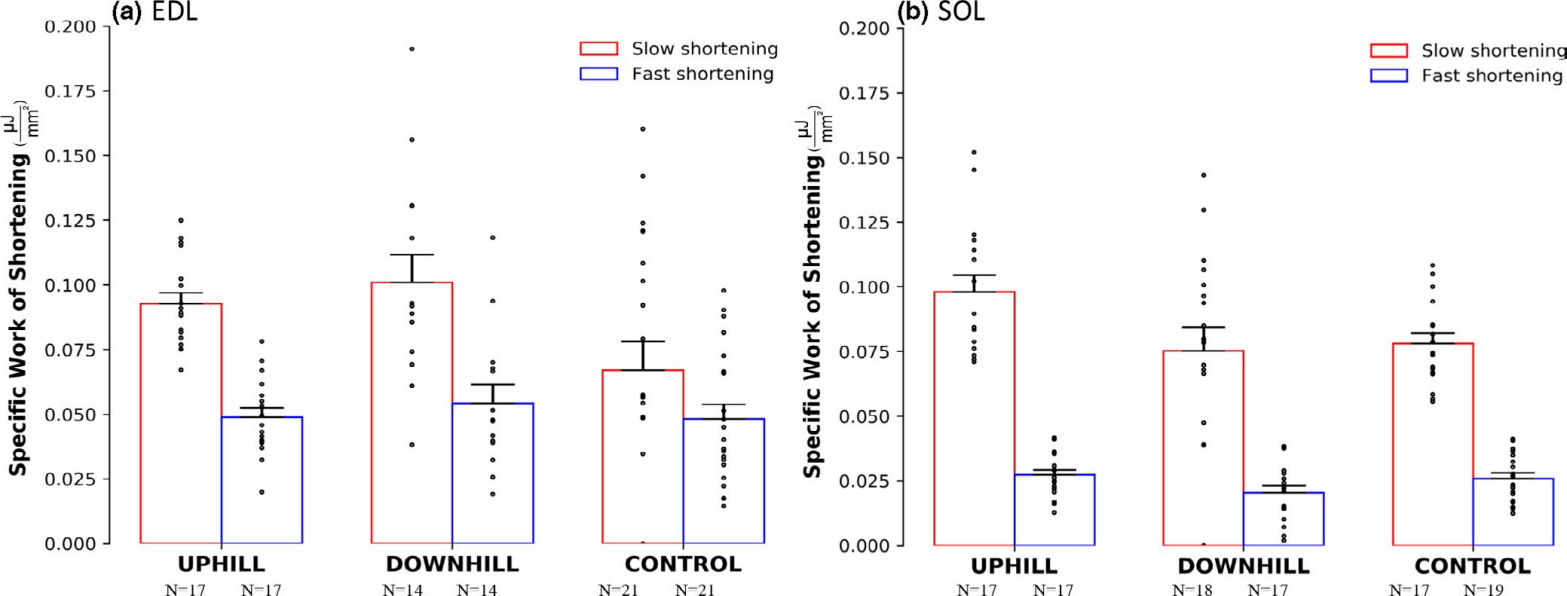
Specific work of shortening in the uphill, downhill, and control groups of the EDL muscle (a) and SOL muscle (b). The red bars represent a slow shortening speed and the blue bars represent a fast shortening speed. The EDL performed 52.3% more work than the SOL during the fast speed, and the EDL in the downhill group performed 46.3% more work than the SOL. On average, both muscles produced 54.1% more work during the slow versus the fast speed. Mean ± SE

For normalized isometric force there was an interaction of group × muscle (F(2) = 5.560, *p* = .005), with no main effects of group or muscle. Within the uphill group, fibers from the EDL produced 18.9% more force than fibers from the SOL (*p* < .001), with no difference between the downhill or control groups. For fibers from the SOL, the uphill group produced 37.4% less force as compared with the downhill group (*p* = .008) (Figure 2), with no difference between groups within the EDL. For the specific work of shortening, there was no interaction of group × muscle × speed, however there was an interaction of muscle × speed (F(1) = 5.936, *p* = .016) and group × muscle (F(2) = 3.571, *p* = .030). There was also a main effect of speed (F(1) = 121.98, *p* < .001) and muscle (F(1) = 16.932, *p* < .001). Fibers from the EDL performed 52.3% more work than fibers from the SOL during the fast speed with no difference between muscles for the slow speed. In the downhill and uphill groups, fibers from the EDL performed 46.3% and 20.8% more work than fibers from the SOL, respectively, with no difference within the control group (Figure 3a,b).

### 3.2 Residual force depression

**FIGURE 4:**
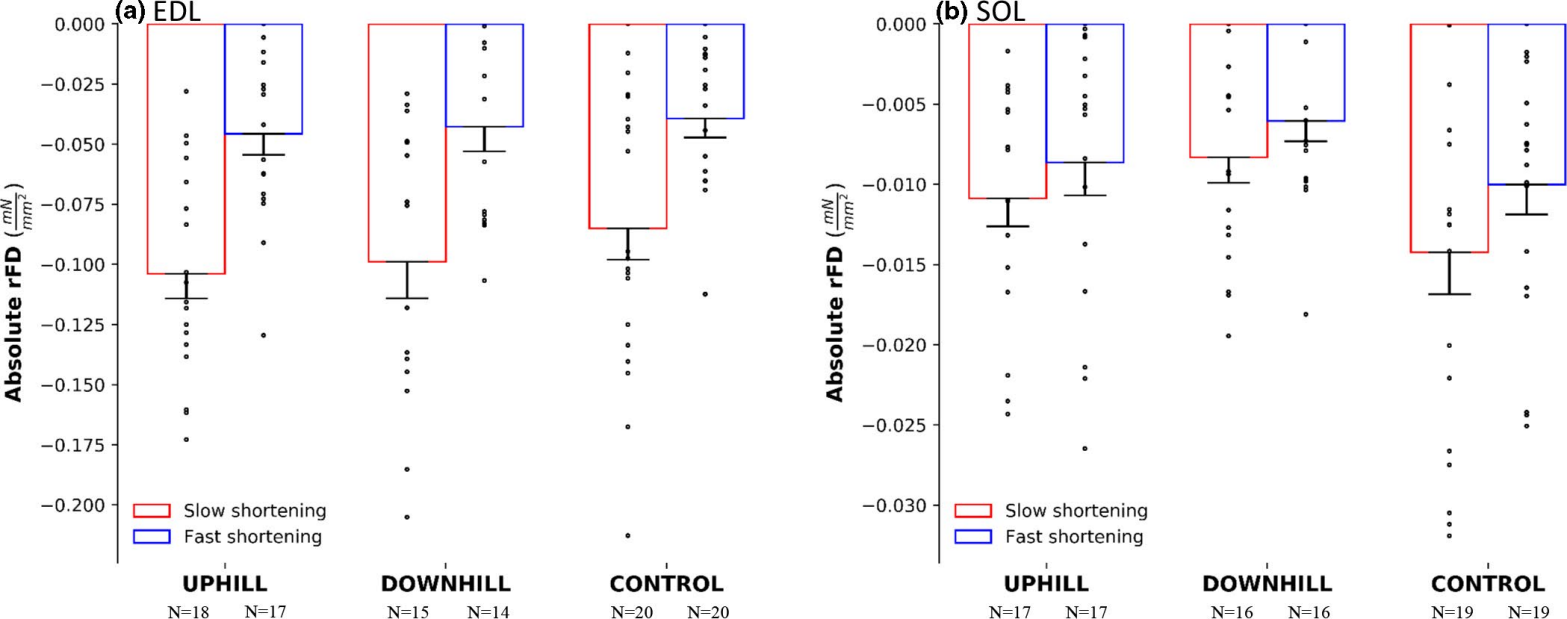
Absolute rFD in the uphill, downhill, and control group of the EDL muscle (a) and the SOL muscle (b). The red bars represent a slow shortening speed and the blue bars represent a fast shortening speed. The EDL had 76.6% and 88.7% more absolute rFD during the fast and slow shortening speeds compared to the SOL respectively. Mean ± SE

**FIGURE 5:**
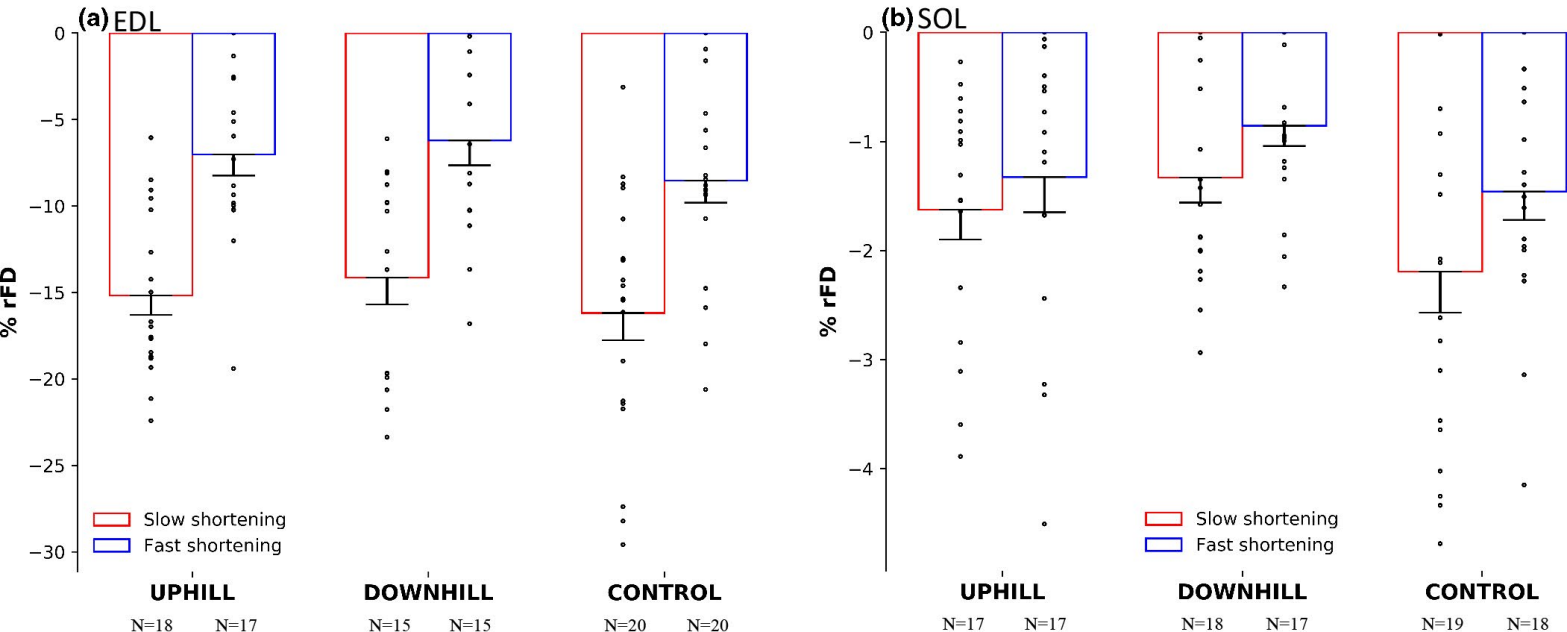
%rFD in the uphill, downhill, and control group of the EDL muscle (a) and SOL muscle (b). The red bars represent a slow shortening speed and the blue bars represent a fast shortening speed. The EDL had 81.1% and 88.7% more %rFD during both the fast and slow speed as compared with the SOL respectively. Mean ± SE

Residual force depression was present in both muscles and all groups (*p* = .030–<.001). For absolute rFD, there was no interaction of group × muscle × speed, however there was an interaction of muscle × speed (F(1) = 36.362, *p* < .00) whereby fibers from the EDL experienced 76.6% and 88.7% more absolute rFD during both the fast and slow speed shortening (Figure 4a) as compared with fibers from the SOL (Figure 4b), respectively. There were main effects for muscle (F(1) = 109.017, *p* < .001) and speed (F(1) = 44.958, *p* < .001). For %rFD, there was no interaction of group × muscle × speed, however there was an interaction of muscle × speed (F(1) = 54.420, *p* < .001), whereby fibers from the EDL experienced 81.1% and 88.7% more %rFD during both the fast and slow speed (Figure 5a) as compared with fibers from the SOL (Figure 5b), respectively. Additionally, fibers from the EDL, but not the SOL experienced 56.6% greater %rFD during the slow compared to the fast shortening speed. As well, there were main effects for muscle (F(1) = 267.426, *p* < .001) and speed (F(1) = 64.394, *p* < .001).

### 3.3 Stiffness depression

**FIGURE 6:**
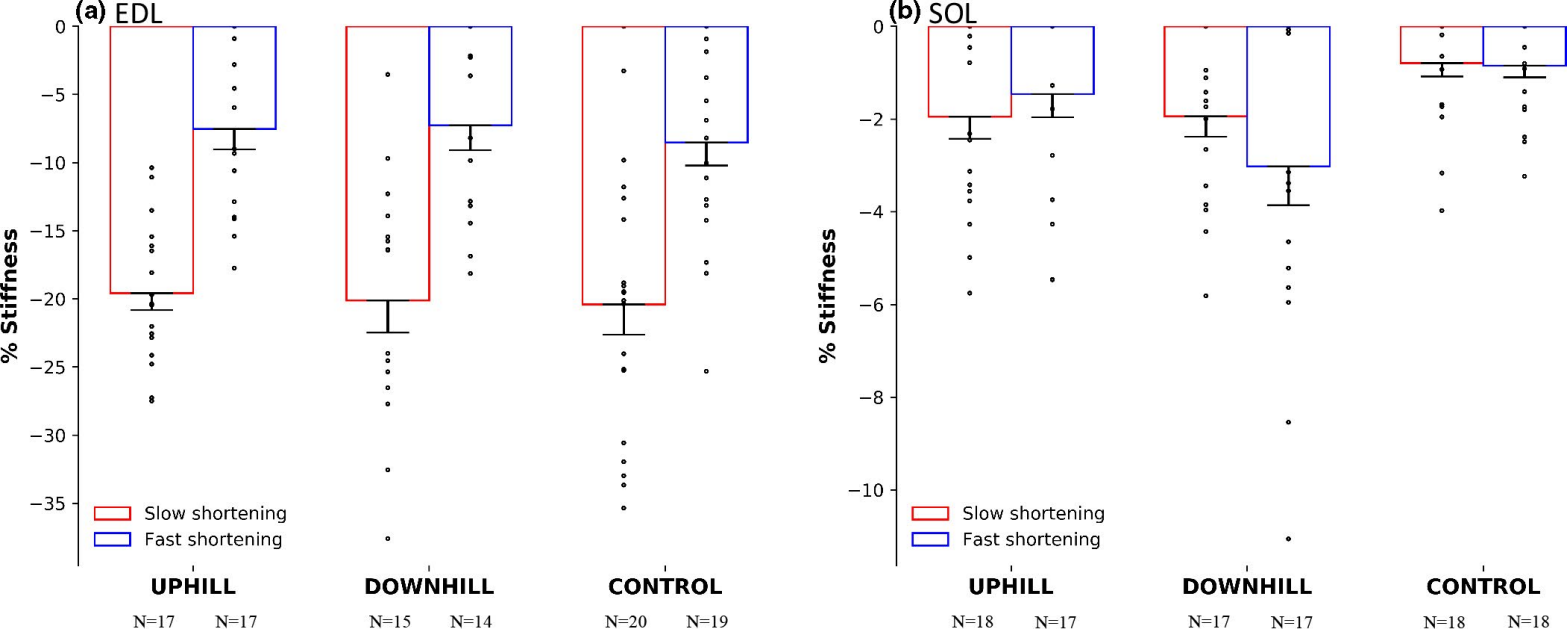
% stiffness depression in the uphill, downhill, and control group of the EDL muscle (a) and the SOL muscle (b). The red bars represent slow shortening speed and the blue bars represent a fast shortening speed. The EDL had 72.3% and 92.2% more stiffness depression during both the fast and slow speed as compared to the SOL respectively. Mean ± SE

There was no interaction of group × muscle × speed, however there was an interaction of muscle × speed (F(1) = 65.543, *p* < .001), whereby fibers from the EDL experienced 77.3% and 92.2% more stiffness depression during both the fast and slow speed (Figure 6a) as compared with fibers from the SOL (Figure 6b), respectively. There were main effects for muscle (F(1) = 240.979, *p* < .001) and speed (F(1) = 61.835, *p* < .001).

### 3.4 Relationship between force depression, work of shortening, and stiffness depression

**FIGURE 7:**
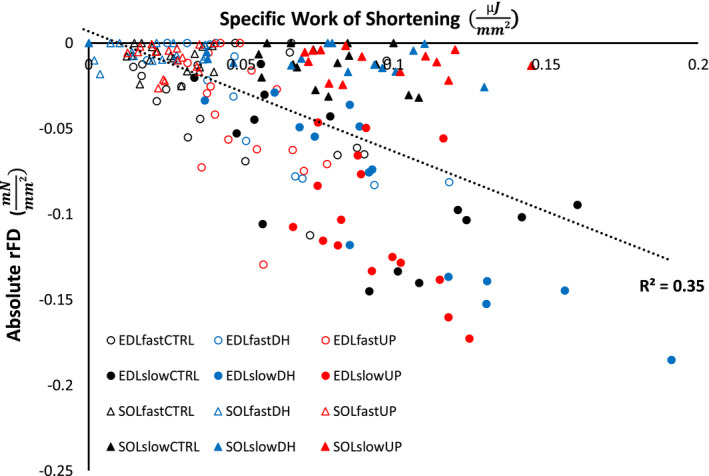
Regression analysis of absolute rFD (y‐axis) vs specific work of shortening (x‐axis). There was a significant relationship between absolute rFD and specific work of shortening, when pooled across muscle, group, and speed (*R* ^2 ^= .35)

There was a significant relationship between absolute rFD and the specific work of shortening when pooled across muscle, group and speed (Figure 7) (*R* ^2 ^= .35, F(1) = 96.220, *p* < .001), indicating that with increasing work there was greater rFD. However, this relationship varied for each condition, the *R* ^2^ values were: EDLfastCTRL, .12; EDLfastDH, .72; EDLfastUP, .23; EDLslowCTRL, .40; EDLslowDH, .82; EDLslowUP, .23; SOLfastCTRL, .026; SOLfastDH, .0024; SOLfastUP, 2.0E^−06^; SOLslowCTRL, .12; SOLslowDH, .12; SOLslowUP, .022. The relationships between specific work of shortening and absolute rFD were very weak for most of these conditions, with the exception of the EDL muscle in the downhill group which showed a strong relationship between specific work of shortening and absolute rFD.

**FIGURE 8:**
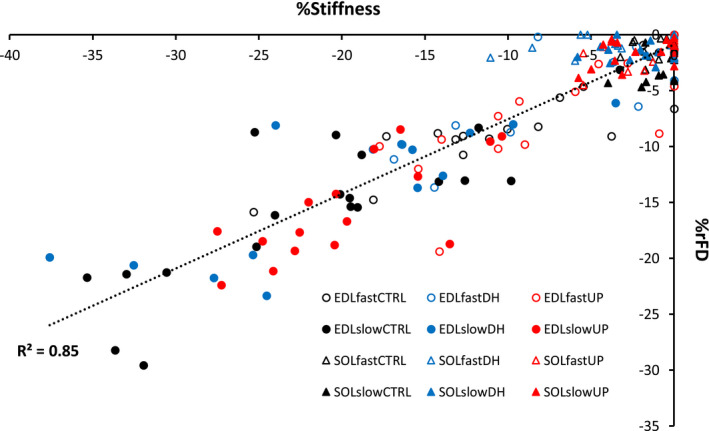
Regression analysis of %rFD (y‐axis) vs %stiffness (x‐axis). There was a strong significant relationship between %rFD and %stiffness when pooled across muscle, group, and speed (*R* ^2 ^= .85)

Additionally, there was a strong, significant relationship between %rFD and %stiffness depression (*R* ^2 ^= .85, F(1) = 1107.268, *p* < .001) when pooled across muscle, group and speed (Figure 8). The increasing %rFD with increasing stiffness depression indicates fewer force producing attached cross‐bridges in the rFD state. For each condition, the *R* ^2^ values were: EDLfastCTRL, .77; EDLfastDH, .70; EDLfastUP, .60; EDLslowCTRL, .68; EDLslowDH, .64; EDLslowUP, .55; SOLfastCTRL, .0018; SOLfastDH, .16; SOLfastUP, .31; SOLslowCTRL, .36; SOLslowDH, .05; SOLslowUP, .22.

## 4 DISCUSSION

We investigated the modifiability of rFD following 4 weeks of uphill and downhill running in rats. In line with our hypotheses, rFD was not different across uphill, downhill and control groups. Single muscle fiber from the fast‐type EDL muscle generated more work during shortening, and experienced greater rFD compared to fiber from the slow‐type SOL muscle. Additionally, greater rFD was observed following the slow shortening contraction as compared with the fast shortening contraction (all of these findings are expanded upon below). We showed strong relationships between stiffness depression and rFD, such that with a greater inhibition of cross‐bridge attachments (i.e., stiffness depression) there was increasing rFD magnitude. Thus, in the present study, this cross‐bridge inhibition based mechanism of rFD appears to be unaltered following training.

### 4.1 Residual force depression and specific work of shortening

The EDL is a faster contracting muscle than the SOL (Goodman et al., 2003). Based on the imposed shortening velocities in the current study, these muscles are operating on different portions of the force‐velocity relationship (Bottinelli et al., 1991) which allowed the fibers from the EDL to perform more work than fibers from the SOL for a given shortening speed, thus incurring greater rFD (Joumaa et al., 2015; Lee & Herzog, 2003; Morgan et al., 2000; Pinnell et al., 2019). There was a tight coupling between rFD and stiffness depression (Figure 8), indicating less attached cross‐bridges in the rFD state (Joumaa et al., 2018; Maréchal & Plaghki, 1979; Minozzo & Rassier, 2013; Rassier & Herzog, 2004). Similarly, Joumaa et al. (2015) found that single muscle fibers from the fast‐type rabbit psoas muscle experienced 1.8 times greater rFD compared to the slow‐type rabbit soleus muscle (Joumaa et al., 2015), which supports our findings as fibers from the fast‐type EDL muscle experienced greater rFD compared to fibers from the slow‐type SOL muscle.

Although our %rFD values for fibers from the EDL (Figure 5a; 11%) matched the literature, the values for the fibers from the SOL were lower (Figure 5b; 1.5%) than the 8%–72% that has previously been reported in vitro (Abbott & Aubert, 1952; Edman, 1975; Herzog & Leonard, 1997; Herzog et al., 1998; Joumaa & Herzog, 2010; Joumaa et al., 2012; Maréchal & Plaghki, 1979; Meijer, 2002; Morgan et al., 2000; Pun et al., 2010; Sugi & Tsuchiya, 1988), and the 5%–39% reported for in‐vivo human data across different muscle groups (Chen & Power, 2019). As rFD is highly dependent on speed of shortening (and thus work) (Herzog & Leonard, 1997; Herzog et al., 2000; Lee et al., 2000), this discrepancy in rFD values could be due to the speeds chosen for these experiments. It is highly likely the slow‐type SOL muscle would have experienced greater rFD if slower speeds were chosen. Fibers from the fast‐type EDL muscle experienced 80% and 89% more rFD compared to fibers from the slow‐type SOL muscle for the fast and slow shortening contractions, respectively. This highlights that the discrepancies in rFD present across fiber types is owing to distinct force‐velocity curves that allow for fast‐type muscles to perform more work during shortening in comparison to slow‐type muscles, for a given speed (Joumaa et al., 2015; Lee & Herzog, 2003; Morgan et al., 2000; Pinnell et al., 2019).

Fibers from both the EDL and SOL muscles performed more work for the slow compared to the fast shortening speed, which was the expected outcome. The difference in work across speeds was larger in the SOL compared with the EDL. The EDL showed a velocity‐dependence for rFD, such that there was greater rFD for the slow shortening contraction compared to the fast shortening contraction, while the magnitude of rFD for the SOL did not differ across speeds. Since the amount of work performed during shortening is one of the main phenomenological drivers of rFD (Chen et al., 2019; Herzog et al., 2000; Joumaa et al., 2017), it was surprising to not observe this velocity‐dependent factor for the %rFD values of the SOL muscle. Perhaps if slower speeds were chosen, greater differences in rFD would have been experienced between the difference speeds in the SOL muscle.

### 4.2 Is rFD modifiable?

Our findings are consistent with the literature as previous studies investigating the modifiability of rFD at the in‐vivo whole human level, and in‐vitro whole muscle level also noted that rFD was not modifiable through training (Chen et al., 2020; Chen & Power, 2019; Hinks et al., 2020). By investigating this property at the single fiber level, we are able to identify that rFD is not intrinsically modifiable at the cellular level, at least given the constraints of the current experimental design. Perhaps an adaptation to training had occurred in the SOL muscle, whereby even though the fibers performed similar amounts of work as the EDL muscle, they experienced less rFD. This indicates that, while rFD did not appear to be altered with training, the SOL muscle may still be adapting by allowing for more work to be performed without the negative consequences of increasing rFD. This adaptation may be linked to changes in actin stiffness or perhaps cytoskeleton stiffness, such that stress‐induced inhibition of cross‐bridge attachments were minimized – an area for future investigations.

### 4.3 Limitations:

Our results regarding fiber‐type differences could have been bolstered by performing a MHC SDS‐PAGE gel analysis following mechanical testing in order to identify the type of individual fibers. This would have allowed for a more accurate binning of groups, although as noted in the methods section we are confident in the results obtained by our study as the EDL and SOL muscles were specifically chosen because of their high proportions of type II and type I fibers, respectively. Additionally, the slow‐type SOL muscle was disadvantaged for force production during both speeds and could have benefited from a slower “slow speed” in order to tease out velocity‐dependent factors for rFD and work production. However, further studies are required to understand the velocity‐dependence of a slow‐type muscle in the modifiability of rFD. Of note, after setting the initial sarcomere length, sarcomere length was not continuously tracked but was visually observed. The current study could have benefited from analyzing sarcomere lengths during contraction to account for non‐uniform sarcomere lengths which are known to influence force production.

### 4.4 Conclusion

Residual force depression was present across all groups, muscles and speeds, however there was no training‐induced change in rFD. Thus, there does not seem to be an effect of uphill and downhill running on the modifiability of rFD in rats. The magnitude of rFD was greater in single fibers from the fast‐type EDL muscle as compared with single fibers from the slow‐type SOL muscle, with greater rFD values during the slow compared to the fast speed conditions. While rFD did not appear to be altered with training, the SOL muscle may have adapted by allowing for more work to be performed without the negative consequences of increasing rFD.

## 5 ETHICS STATEMENT

All procedures were approved by the Animal Care Committee of the University of Guelph.

## CONFLICT OF INTEREST

No conflicts of interest, financial or otherwise, are declared by the authors.

## Data Availability

Individual values of all supporting data are available upon request.
